# Noise propagation with interlinked feed-forward pathways

**DOI:** 10.1038/srep23607

**Published:** 2016-03-31

**Authors:** Surendhar Reddy Chepyala, Yi-Chen Chen, Ching-Cher Sanders Yan, Chun-Yi David Lu, Yi-Chun Wu, Chao-Ping Hsu

**Affiliations:** 1 Bioinformatics Program, Taiwan International Graduate Program, Institute of Information Science, Academia Sinica, Taipei, Taiwan; 2 Institute of Chemistry, Academia Sinica, Taipei, Taiwan; 3 Institute of Biomedical Informatics, National Yang-Ming University, Taipei, Taiwan; 4 Department of Chemistry, National Taiwan University, Taipei, Taiwan; 5 Genome and Systems Biology program, National Taiwan University, Taipei, Taiwan; 6 Institute of Molecular and Cellular Biology, National Taiwan University, Taipei, Taiwan; 7 Department of Life Science, National Taiwan University, Taipei, Taiwan; 8 Center for Systems Biology, National Taiwan University, Taipei, Taiwan; 9 Research Center for Developmental Biology and Regenerative Medicine, National Taiwan University, Taipei, Taiwan

## Abstract

Functionally similar pathways are often seen in biological systems, forming feed-forward controls. The robustness in network motifs such as feed-forward loops (FFLs) has been reported previously. In this work, we studied noise propagation in a development network that has multiple interlinked FFLs. A FFL has the potential of asymmetric noise-filtering (i.e., it works at either the “ON” or the “OFF” state in the target gene). With multiple, interlinked FFLs, we show that the propagated noises are largely filtered regardless of the states in the input genes. The noise-filtering property of an interlinked FFL can be largely derived from that of the individual FFLs, and with interlinked FFLs, it is possible to filter noises in both “ON” and “OFF” states in the output. We demonstrated the noise filtering effect in the developmental regulatory network of *Caenorhabditis elegans* that controls the timing of distal tip cell (DTC) migration. The roles of positive feedback loops involving *blmp-1* and the degradation regulation of DRE-1 also studied. Our analyses allow for better inference from network structures to noise-filtering properties, and provide insights into the mechanisms behind the precise DTC migration controls in space and time.

Most of the cellular processes, which are various biochemical reactions, are inherently “noisy” because of extrinsic and intrinsic fluctuation of various factors. Even in isogenic populations under identical environmental conditions, the cells may show greatly different phenotypes^1^,^2^,^3^,^4^. Gene expression can be highly noisy^1^,^4^, partly due to the burst production in mRNA and proteins, and thereby leading to a large cell-to-cell variations^5^,^6^,^7^. The expression of a gene in one cell can be affected by its upstream noise, other global factors, as well as its own intrinsic noise in the expression^8^. Noise can be both an obstacle for some types of cellular functions^9^,^10^,^11^ as well as a useful feature for others^12^,^13^,^14^,^15^,^16^,^17^,^18^. Living organisms go through a sequence of decision-making checkpoints that can not be reversed. Thus, cells need ways to cope with those fluctuations. Given the high level of stochastic fluctuations in gene expression at the intracellular level^1^,^4^ it is hard to imagine that stability can be achieved without certain endogenous regulatory mechanism, such as feedback or feed-forward controls^19^,^20^. Understanding how cells efficiently and correctly process information in noisy environments is of fundamental importance.

In development, organisms grow with the same spatial and temporal patterns, with few variations among individuals. How the precise developmental events are controlled under the noisy condition has been an important question to answer^21^,^22^. Gene regulation networks are often composed of a small set of recurring interaction patterns called network motifs^23^,^24^. Many motifs perform specific dynamic functions (as reviewed in ref. 25). In the cases studied so far, these motifs seem to preserve their autonomous functions even in their natural contexts, wired into the regulatory networks of the cell^25^,^26^. Therefore, studying the dynamics and fluctuations of biological processes with one particular network may help us to understand many other systems with networks composed of similar motifs.

Among the network motifs in biological systems, feed-forward loops (FFLs) play a significant role^27^. All possible FFL architectures have been identified and many were shown to regulate a multitude of cellular processes^23^,^24^,^25^,^27^ in a diverse range of organisms, from bacteria to human cells^28^,^29^,^30^,^31^,^32^,^33^,^34^. The regulatory interactions in FFL can be positive (activation) or negative (repression). On the basis of the effects acting on the downstream node in the two pathways, FFLs are classified as coherent or incoherent. A coherent FFL (cFFL) is capable of filtering noise asymmetrically (i.e., only in one of the gene regulatory states, either ON or OFF state)^25^,^27^,^35^. However, to have a precise and robust phenotype of a particular trait (or cellular function), the noise must be controlled in both ON and OFF-states of the gene. The cFFLs often combine with other FFLs or other motifs and form interlinked FFLs (IFFL) or other more complex circuits, and the noise-filtering property in these interlinked networks may be improved. The consequences of combined network motifs in terms of noise control need to be studied. To the best of our knowledge, such a study of combined IFFL has not been reported. In the present work, we analyze the general noise-propagating properties in a development gene regulatory network of *C. elegans*, to understand the link between network structure and its properties in robustness and noise-resisting.

In *C. elegans*, its reproductive organ, the hermaphrodite gonad, is shaped by migration of the two distal tip cells (DTCs) located on the anterior and posterior ends of the gonad. The final U shape of hermaphrodite gonad arms is determined by a sequential three-phase migration of these two DTCs. The DTCs undergo a long-range migration and re-orient twice, providing a paradigm for studying the spatiotemporal regulation of cell migration *in vivo*^36^. The timing of the first dorsalward DTC turning is regulated by a set of genes: *blmp-1*, *lin-42*, *daf-12*, *lin-29* and *dre-1*^37^,^38^,^39^,^40^. DAF-12 is a steroid hormone receptor^41^; LIN-29 and BLMP-1 are both zinc-finger transcription factors^37^. Both LIN-29 and BLMP-1 are regulated by the Period-like protein LIN-42 at the transcriptional level^39^. The F-box protein DRE-1 promotes BLMP-1 degradation via the ubiquitin-mediated proteasome pathway^42^. A single mutation in *daf-12*, *lin-29* and *dre-1* does not affect DTC migration; however, double mutations delay the L3-specific DTC migration pattern, which frequently fails to take place, even in the L4 or adult^37^. In contrast, mutants defective in *blmp-1* or *lin-42* show a precocious DTC migration pattern^37^,^39^. It is observed that DRE-1 and DAF-12 prevent BLMP-1 expression in late L3 stage^37^. However, when *blmp-1* and *daf-12* mutations are combined, the DTC migration is heterogeneous^37^ and this result indicates susceptibility to variations in individual worms. The molecular basis of the heterogeneous phenotypes is unclear.

From previous observations, one can construct a gene regulatory network that includes steroid hormone signaling (DAF-12), gene transcription (LIN-29, BLMP-1, LIN-42) and protein degradation (DRE-1), in the control of the L3-specific DTC migration pattern in *C. elegans*. This gene regulatory network controls the timely expression of the netrin guidance receptor UNC-5, which is both necessary and sufficient for DTC dosral migration^38^. While BLMP-1 inhibits *unc-5* transcription, LIN-29 and DAF-12 promotes *unc-5* transcription^37^. To understand the temporal regulation and noise-filtering effect of components in the regulatory network, we constructed a mathematical model describing the time change in protein levels. The model contains three input nodes (*daf-12, lin-42* and *dre-1*), two intermediate nodes (*lin-29* and *blmp-1*) and one output node (*unc-5*) (Fig. 1a). The timing of UNC-5 expression should be constrained into a specific developmental time window, neither too early nor too late. The noises in the upstream genes may propagate to UNC-5 and affect the timing. There are also two positive feedback loops (PFLs) coupled with the network. The presence of interlinked cFFLs and positive feedbacks in Fig. 1a prompted us to evaluate their potential roles in noise-filtering in the network. In this work, we studied the *unc-5* gene regulation network for the noise-filtering property, with a stress on the roles of interlinked network motifs such as cFFLs and PFLs.

## Results

### Role of interlinked Feed-Forward Loops (IFFL) in noise-filtering

FFLs are network motifs that contain direct and indirect pathways from input to output nodes. When the input gene is switched, there is a delay in the indirect pathway. In the coherence case, depending on how the output gene integrates the two signals, the switch in the output gene may be delayed, and the time-delay provides a chance to filter a fluctuation in the upstream, or the noise^25^,^27^. However, such a noise-filtering effect is asymmetric, which is only effective in the ON or OFF-state of the gene.

The DTC migration network contains multiple pathways from input to output, forming more than one FFL. Here we postulate that these IFFL further help to reduce the input noise in both ON and OFF-states. In other words, the IFFL in the current DTC network may be able to filter the propagated noise from DAF-12 and LIN-42 to UNC-5, which we tested and discuss in this section.

**Figure 1:**
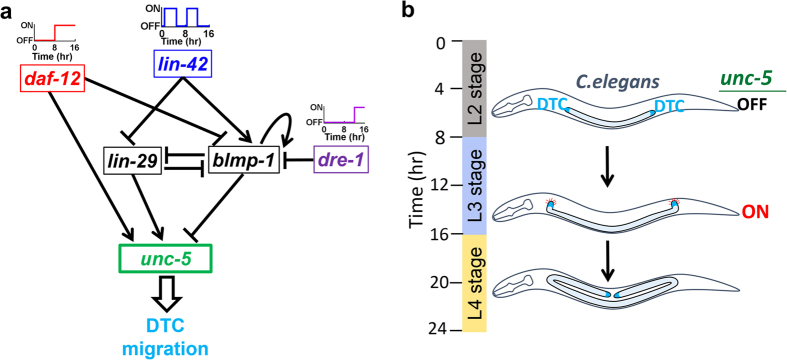
(**a**) Regulatory network for the DTC migration in *C. elegans*. (**b**) Schematic representation of DTC migration in *C. elegans* and its timing.

The DTC migration regulation network we studied is shown in Fig. 1a. *daf-12*, *lin-42* and *dre-1* were included as input nodes with their temporal activity indicated. The regulations are described by the Hill function (with further details in Methods). When more than one upstream gene regulates a downstream gene, the combination of the corresponding Hill functions are similar to the Boolean AND or OR operations. The logic gates were determined based on experimental or computational observations and the same logic combinations were used when analyzing the subnetworks (see Methods). To study the noise-filtering effect in DTC migration, we used a stochastic Langevin’s equation to simulate the gene regulatory network. We introduced the noise, which includes the burst effect in protein production in a modified Langevin equation (Supplementary of ref. 43, with further details in Methods). We simulated the deterministic and stochastic trajectories of the current network as shown in Fig. 1a.

Since we aimed at demonstrating the noise-filtering properties in structure of the network, we set up tests with a good number of general parameter sets with least screening. For most of the tests presented in this work, we randomly selected 1000 parameter sets out of 3.08 × 10^7^ of those produced downstream nodes (UNC-5, BLMP-1 and LIN-29) expression as in wild type. For tests related to *daf-12* and *lin-29* mutants, we randomly chose 1000 parameter sets that produced wild type and UNC-5 expression as in six homogeneous mutants, as there were 5.35 × 10^6^ such parameter sets. For simulating the heterogeneous *blmp-1;daf-12* mutant, we used more restricted parameter sets that reproduce the phenotype of the wild type, six homogeneous mutants and the heterogeneous *blmp-1;daf-12* mutant in a stochastic simulation, and there were 109 parameter sets.

The magnitude of noise was calculated as the Fano factor (FF), defined as the variance divided by the mean value of the component, and the distribution of FF was reported. The FF of an intrinsically noisy protein is typically 17 from our parameter settings (see Methods). To study propagated noises, the noise of upstream genes was added, whereas the noise in the downstream components was set to zero, and the FF was calculated from the fluctuation due to the upstream genes.

### An IFFL filters noises from DAF-12

**Figure 2:**
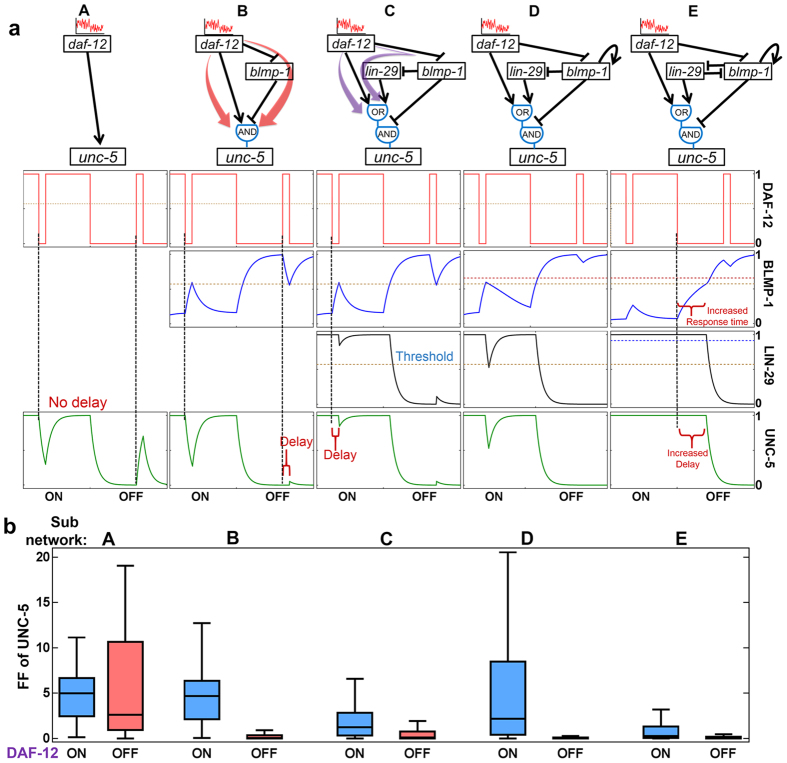
Noise filtering in IFFL. (**a**) Schematic representation of noise filtering for a spike in the OFF and ON-states through FFL and IFFL (subnetworks A-C) and feedbacks (subnetworks D and E). (**b**) Propagated noises from DAF-12 as observed fluctuation in UNC-5 in different subnetworks as shown on the top. Intrinsic noises were added to DAF-12 only, and the FF of the propagated fluctuation in UNC-5 are calculated and plotted. Blue boxes are for DAF-12 sampled in its ON-state, while red boxes are for the OFF-state. Shown are box plots for the FFs for UNC-5 from the 1000 sampled parameter sets, with the median, and the upper and lower quantiles indicated. Whiskers indicate furthest observation within 1.5 times the interquartile range outside of the upper and lower quartiles. We excluded the outliers for better visibility.

We first studied the noise originating from DAF-12 in effecting UNC-5, the output of the network that controls DTC turning. In Fig. 2, we depict several subnetworks that can propagate signals from *daf-12* to *unc-5*. As seen in Fig. 2a, with subnetworks originated from *daf-12* input, we schematically illustrate the noise-filtering property. Direct regulation from *daf-12* to *unc-5* (subnetwork A) does not show any delay downstream, and the DAF-12 noise is propagated to UNC-5, regardless of its activity. According to the classification in ref. 27, Subnetwork B is a type-4 cFFL with an AND logic gate, with a delayed response in turning the input to ON. It is capable of filtering transient spikes in the OFF-state but not in the ON-state. In subnetwork C, *lin-29* and its associated pathways are added; it forms a generalized FFL (purple color) that starts with *daf-12*, with *blmp-1* and *lin-29* as the intermediate nodes, and ends at *unc-5*. It is similar to a type-1 cFFL with an OR gate^27^, which shows a delayed response when the input turns OFF, filtering the ON-state noise. Thus with multiple pathways, IFFL as in subnetwork C can buffer the noise in both ON and OFF-states of input genes.

With stochastic numerical simulation, we further demonstrate the noise-filtering capability in the IFFL, subnetwork C. As seen in Fig. 2b, results for subnetwork A, a direct regulation from *daf-12* to *unc-5* sets the basal level of noise propagation. By adding *blmp-1* to it, subnetwork B can reduce the OFF-state but not the ON-state noise from DAF-12. The ON-state noise is also reduced when *lin-29* is added downstream of *blmp-1* and a positive regulator for *unc-5*, forming subnetwork C. The stochastic simulation results also show that IFFL as subnetwork C can indeed buffer the DAF-12 noise in both OFF and ON-states. Therefore, adding layers of feed-forward and forming IFFL helps the system better filter upstream noises.

### An IFFL filters noises from LIN-42

**Figure 3:**
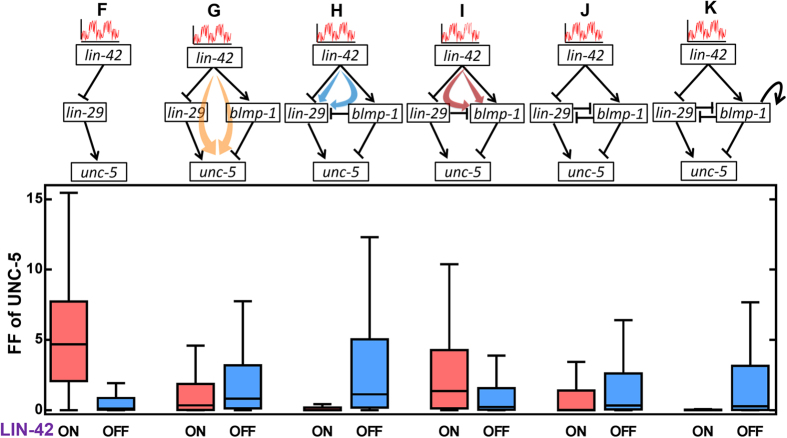
Propagated noise from LIN-42 as observed fluctuation in UNC-5 in different subnetworks indicated on the top. Intrinsic noise was added to LIN-42 only, and the FF of the propagated fluctuation in UNC-5 is calculated and plotted. Red boxes are for LIN-42 sampled in its ON-state, which also corresponds to the BLMP-1 ON-state, LIN-29 OFF-state, and UNC-5 ON-state. Blue boxes are for the opposite state. Other details are similar to that in Fig. 2.

The subnetworks we studied, starting from *lin-42* to *unc-5* are shown in Fig. 3. A direct pathway through *lin-29* (subnetwork F) shows how noise propagates to UNC-5. The signal can also propagate through *blmp-1*, adding an interaction mediated by *blmp-1* (subnetwork G), and forming a generalized cFFL among the four components. The feed-forward structure in subnetwork G can be regarded as a generalized form of cFFL types 2 or 3 with an AND gate. Both of these cFFLs exhibit a delayed response in turning OFF^27^, and thus, they are potentially able to filter LIN-42 ON-state noise as long as the time needed to pass through these two pathways are not the same. As shown in Fig. 3, for subnetwork G, the LIN-42 ON-state noise is reduced.

*lin-29* and *blmp-1* inhibit each other, and these inhibitions also offer a chance to feed-forward the signal, in additional to the feedback effect that is discussed in the following section. In subnetwork H, we include the inhibition from *blmp-1* to *lin-29* and further study the IFFL effects. An additional type 3 cFFL with an AND gate (marked in light blue color) is formed between *lin-42*, *blmp-1* and *lin-29*, which reduces the ON-state propagated noise from *lin-42* to *lin-29*, thereby leading to reduced propagated noise in UNC-5 (Fig. 3). As shown in subnetwork I, including the inhibition from *lin-29* to *blmp-1*, a type-4 cFFL (marked in light brown) with an AND gate formed between *lin-42*, *lin-29* and *blmp-1* is formed, which filters the LIN-42 OFF-state noise. Subnetwork J includes the both the FFLs shown in subnetworks H and I and is able to filter the noise in both ON and OFF-states of LIN-42, with a mild increase in both the states as compared to the well-filtered ON-state for H or the OFF-state for I. Our results show that the both ON and OFF-state noises in LIN-42 are buffered and this noise-buffering effect can be understood through layers of FFLs in the subnetworks. Similar behavior is also observed from the subnetworks from DAF-12. Therefore, the noise-filtering property of an IFFL can be largely derived from that of the individual FFLs.

### Positive feedback loops in filtering the propagated noise

PFLs can buffer the propagated noise^44^,^45^,^46^. Computational study of PFLs that maintain signal sensitivity showed PFLs with mutual activation can buffer the noise in both OFF and ON-states as compared to PFLs with mutual repression^45^. However, feedback motifs in natural systems are not isolated, and they often coupled with additional PFLs^46^,^47^,^48^,^49^. Because PFLs with mutual inhibition and mutual activation have different strengths (roles) of noise-buffering in the ON and OFF-states^45^, we further aimed to investigate the role of the two coupled PFLs in noise-filtering.

Two PFLs exist in the DTC network: one is the autoregulation of *blmp-1* (auto-PFL), and the other is the mutual repression between *lin-29* and *blimp-1* (subnetworks D and E, respectively, in Fig. 2). In the current network, both the PFLs are integrated with other FFLs. Hence, they are regulated by upstream genes and regulating downstream genes. Adding auto-PFL on *blmp-1* reduced the propagation of DAF-12 OFF-state and LIN-42 ON-state noise in UNC-5 (subnetwork D in Fig. 2, subnetwork K in Fig. 3). Results for subnetwork E, with an additional positive feedback of mutual repression, also show reduced noise in the DAF-12 ON-state.

Positive feedback increases response time and thus provides a better averaging over rapid fluctuations in both the ON and OFF-states^44^,^45^. In the current study, auto-PFL on BLMP-1 acts with other regulators with an OR gate, which lead to an increases of response time in the BLMP-1 ON-state but not in the OFF-state. The asymmetric response time change of BLMP-1 implies noise-filtering in the ON-state of BLMP-1 or the OFF-state of DAF-12 (subnetwork D in Fig. 2). In contrast, another PFL with mutual repression is integrated with an AND gate on BLMP-1, increases the response time in both ON and OFF-states (subnetwork E in Fig. 2), and the propagated noise is filtered in both states. Therefore, the noise-filtering effects of PFL, when coupled to other regulations in a network, is dependent on the logic gate that combines the output of PFL and other inputs. In the present study, logic gates of the two PFL are determined by the robustness in reproducing the wild type and homogeneous mutants (Supplementary Table S1). It is interesting to see the logic gate setting that generates desirable phenotypes with the highest probability also allows good noise filtering.

Additional results are included in Supplementary Fig. S1, where we show the magnitude of propagated fluctuation at BLMP-1 and LIN-29. Again we observed that auto-PFL on *blmp-1* alone was sufficient for filtering the propagated noise from DAF-12 and LIN-42 when BLMP-1 is in the ON-state (Supplementary Fig. S1).

### Effect of intrinsic noises in BLMP-1 and LIN-29

Every gene expression is stochastic in nature. In this part, we studied the effect of intrinsic noise of intermediate nodes, BLMP-1 and LIN-29.

Both *blmp-1* and *lin-29* are coupled to PFLs in the network. For *blmp-1*, an upstream inhibitor DAF-12 becomes active in the early L3^37^,^41^, whereas the activator, LIN-42, oscillates in its expression relative to the developmental molts in *C. elegans*^39^. Moreover, immunostaining detected BLMP-1 during L2 and early L3 but not late L3 stage^37^. Therefore, the expression of *blmp-1* needs to be maintained until early L3 stage, including the late L2 stage when the activator of *blmp-1*, LIN-42, is in the low-expression phase of the oscillation. The auto-positive regulation of *blmp-1* was necessary for maintaining its expression^37^. *lin-29* mutant is the wild type in DTC turning, which implies that it is the auto-PFL, not the mutual inhibition involving LIN-29, that maintains the activity of BLMP-1. While searching for parameters that produce wild-type expression in UNC-5, BLMP-1 and LIN-29, we found that the most of the selected parameter sets have a low threshold level for BLMP-1 auto-activation (Supplementary Fig. S3), indicating a strong auto-activation in BLMP-1.

PFL has been reported to amplify intrinsic noise^25^. Similar results were also observed in our system, with auto-PFL increasing the intrinsic noise (Supplementary Fig. S2, subnetworks T and K). While BLMP-1 is in the ON-state, the strong auto-PFL helps maintain the ON-state. However, in the OFF-state, because of the low auto-activation threshold, intrinsic noise allows the BLMP-1 to reach to a higher steady state and thereby increasing the OFF-state noise. Therefore, the auto-PFL of *blmp-1* amplifies the intrinsic noise at the OFF-state of BLMP-1 because of its spurious activation. On the other hand, the PFL from mutual inhibition only mildly increased the intrinsic noise at BLMP-1 and LIN-29 (Supplementary Fig. S2, subnetwork J).

**Figure 4:**
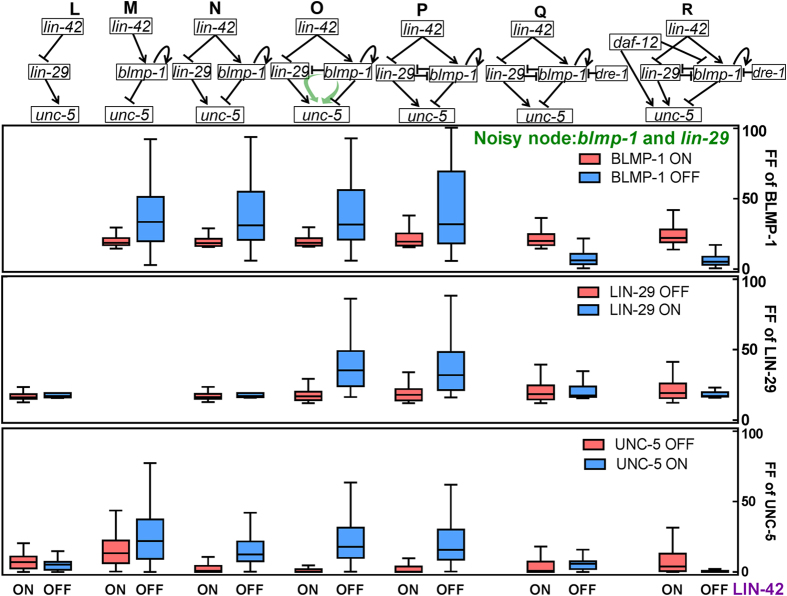
Regulation of noise propagation from BLMP-1 and LIN-29 to UNC-5. Shown are the subnetworks analyzed, and the FF distribution for BLMP-1, LIN-29 and UNC-5. Blue boxes are for LIN-42 OFF-state, which also corresponds to the BLMP-1 OFF-state, LIN-29 ON-state and UNC-5 ON-state. Red boxes are for the opposite state. Other details are similar to that in Fig. 2.

We next studied how the noises in BLMP-1 and LIN-29 may be propagated to UNC-5. Both are direct upstreams of UNC-5, regulating *unc-5* through an AND logic, and it allows better noise filtering in the OFF-state noise of UNC-5. We allowed *blmp-1* and *lin-29* nodes to be noisy and observed the noise propagation in different subnetworks as shown in Fig. 4. As compared with direct regulation from *lin-29* and *blmp-1* (subnetworks L, M) when combining both regulators (subnetworks N, O and P), the OFF-state noise of UNC-5 was generally reduced (which corresponds to the ON-state of LIN-42 shown in the figure).

DRE-1 functions in the SCF ubiquitin ligase complex and post-translationally regulates BLMP-1 by degrading it^42^. Its expression in the mid L3 stage reduces BLIMP-1 level and allows DTC turning. When DRE-1 is expressed (subnetwork Q), it keeps the BLMP-1 at a lower level, and the degradation by DRE-1 reduces BLMP-1 OFF-state noise, leading to a low noise level in UNC-5 as well (OFF-state of LIN-42).

In subnetwork R, it is seen that adding *daf-12* regulation reduces the LIN-29 ON-state noise and further reduces the UNC-5 ON-state noise with a mild increase in OFF-state noise. This effect is similar to that observed in subnetwork C (Fig. 2). In maintaining the UNC-5 ON-state, either *daf-12* or *lin-29* presence is sufficient, and thus the UNC-5 ON-state noise is reduced (shown as LIN-42 OFF-state in Fig. 4).

### Network architecture controls the overall noise of UNC-5

**Figure 5:**
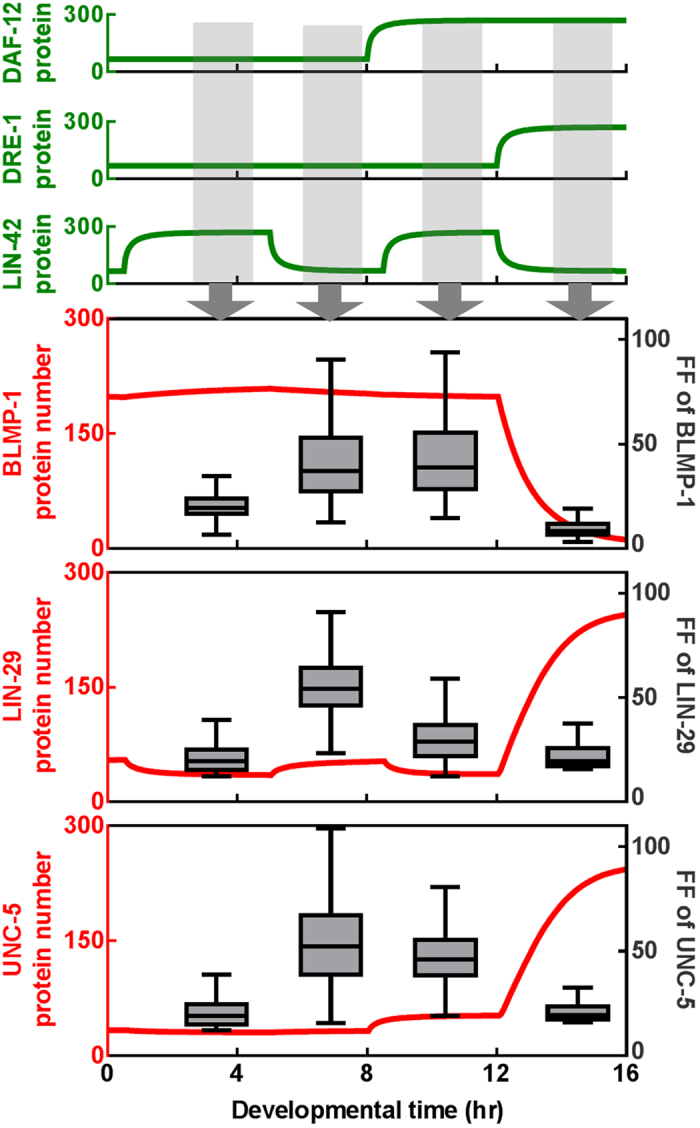
Noise propagation in the complete network, calculated with intrinsic noises included in all nodes. Shown on top are the activity of the three input genes, DAF-12, DRE-1 and LIN-42. The red curves are the mean trajectories, scaled to the left, of the 3 downstream components. Gray boxes and whiskers are for the FF distribution of the corresponding components, calculated at the steady state of the 4 different developmental stages as indicated.

In the current network, BLMP-1 is temporally regulated by *daf-12*, *lin-42*, *lin-29* and *dre-1* genes^37^. From previous results, we can see that UNC-5 noise is well-controlled by IFFL and PFLs. To see the UNC-5 noise level at different developmental time periods, we simulate the complete network shown in Fig. 1a, allowing for all nodes with intrinsic noise and all noise propagation. The result is shown in Fig. 5; at the time of DTC turning, the noise of UNC-5 is kept low, close to its intrinsic noise with an FF of 17. This result shows that the regulation network gives UNC-5 a timing control in turning it on but also in a stringent manner such that the propagated noise is minimal.

In the earlier stages, the noise of UNC-5 varies. The first stage we studied, early L2, has a low level of noise, with LIN-42 turned ON, while DAF-12 and DRE-1 are OFF. In this stage the network is working coherently and UNC-5 is kept in the OFF-state. The second stage is when the oscillatory LIN-42 turned OFF. At this stage, BLMP-1 is maintained in its ON-state through its auto-PFL, and the DRE-1 OFF-state noise can degrade the BLMP-1, thereby leading to the observed noises in BLMP-1 and UNC-5. At the third stage, LIN-42 is turned back ON again, and at about the same time, DAF-12 is now turned ON, sending an inhibitory effect to *blmp-1*. The inconsistent inputs of DAF-12 and LIN-42 lead to a compromised level in the *blmp-1* regulation. In other words, the combination of Hill functions of DAF-12 and LIN-42 in controlling *blmp-1* is in an intermediate level, leading to better noise propagation, and results in a higher noise in BLMP-1. As we have seen in subnetwork Q, R in Fig. 4, adding *dre-1* and *daf-12* regulation decreased the UNC-5 ON-state noise with the price of a mild increase in OFF-state noise. Thus the network architecture play a significant role in expression of a key regulatory protein, UNC-5, with minimal noise.

However, the higher noise in UNC-5 in the earlier developmental stages might cause problems in development. We next studied the dynamics in UNC-5 in light of phenotypes observed in mutants.

### Noise propagation in mutants with homogeneous phenotypes

Single mutants including *lin-29* and *daf-12* shows a wild type DTC turning phenotype^37^. These results imply that the positive regulation of LIN-29 or DAF-12 alone is sufficient to express UNC-5^37^. We further studied the noise-filtering capacity of UNC-5 expression in the two mutant networks. For this test, we selected the parameters that produce the wild type and six other homogeneous mutants.

**Figure 6:**
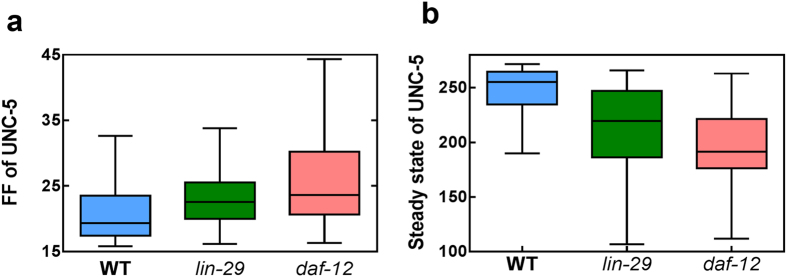
Comparison of UNC-5 noise (**a**) and mean level (**b**) in the wild type, *lin-29* and *daf-12* mutants at the time of DTC turning.

From Fig. 6a, we can see that the noise of UNC-5 during the late L3 stage (at the time of DTC turning) was increased in *lin-29* and *daf-12* mutants. Deterministic simulation in these two mutants shows that UNC-5 expression is similar to that of the wild type, where a clear separation is observed between the ON and OFF-state of UNC-5. However, in these mutants, we observed a mild decrease in steady state level of UNC-5 when it is turned ON in late L3 stage (Fig. 6b). Such a decrease in UNC-5 expression was previously observed in experiments^37^, which supports our simulation results. With a general parameter setting, we show that a partial loss of function in the network could still maintain the desirable outcome (such as UNC-5 expressed at the right time), but the system becomes more fragile due to the higher noise.

### Noise causes the heterogeneous phenotype in the *blmp-1;daf-12* mutant

Noise is the main source of heterogeneity in identical cells^1^,^2^. Although most of the single and double mutants of the DTC migration network show a homogeneous phenotype, *blmp-1;daf-12* mutant shows a heterogeneous phenotype, where the DTC turns both precociously and similar to the wild type (Table 1)^37^.

**Figure 7:**
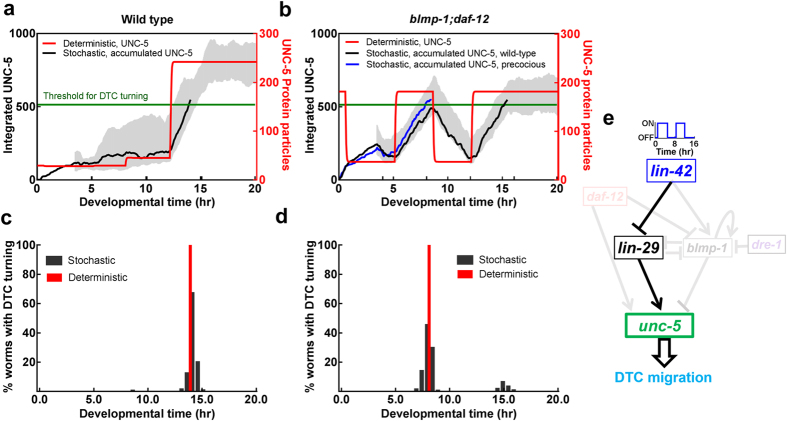
Trajectories and phenotypes for the wild type and *blmp-1;daf-12* mutant. Shown in (**a**,**b**) are deterministic UNC-5 trajectory (red, scaled to the right) and representative stochastic trajectories of accumulated UNC-5 signal (gray lines with one trajectory shown in black) scaled to the left, for the wild type (**a**) and *blmp-1;daf-12* mutant (**b**). In (**b**) a precocious turning trajectory is shown in blue. The DTC turning time distribution is shown in (**c**) for the wild type and in (**d**) for *blmp-1;daf-12* mutant. The network for *blmp-1; daf-12* mutant is included in (**e**).

Deterministic simulation showed that DTC turning is precocious in *blmp-1;daf-12*. When LIN-42 is turned OFF in L2, without BLMP-1’s inhibition, UNC-5 is turned ON (the red trajectory in Fig. 7b) and DTC is allowed to turn early (Fig. 7d).

To demonstrate the noise driven heterogeneity in *blmp-1;daf-12*, we applied stochastic simulation. To correlate the noisy UNC-5 expression with the DTC turning time, we integrated the UNC-5 protein signal and considered that DTC was turned when the signal crossed the threshold value (Fig. 7a,b, with gray trajectories as integrated UNC-5 strength and green line for the threshold). Here we have assumed that DTC turning takes place in a slow time-scale with many down-stream changes following UNC-5, and therefore, an accumulation of UNC-5 signal is accounted.

In the wild type, all the integrated UNC-5 protein signal crossed the threshold value during the late L3 stage (Fig. 7a), and the distribution of DTC turning time was close to that from deterministic simulation (Fig. 7c). In the *blmp-1;daf-12* mutant, the decrease in LIN-42 expression allows UNC-5 to express in the late L2 stage, and thus, UNC-5 expresses precociously, but without the activation from DAF-12, it is expressed at a lower level (Fig. 7b). As a result, many trajectories cross the threshold value precociously (with a typical blue color trajectory shown in Fig. 7b), but about 10% of the worms with integrated UNC-5 signal do not reach the threshold value until late L3 (a representative trajectory shown in black), thereby showing wild-type phenotype. Therefore, *blmp-1;daf-12* mutant expresses a relatively high level of UNC-5 in the L2 stage, but not sufficient to make every DTC turn. Our computational study showed how the dynamics and noises in the UNC-5 lead to the heterogeneity in the phenotypes.

## Discussion

Robustness of different cellular functions may come from the local structures of the regulatory network, or motifs, such as FFLs. Coherent FFLs provide a time delay in the two regulating pathways, which offers a chance to filter ON or OFF-state input noise. In the present work, we provide insight into how multiple FFLs or IFFL are integrated in a developmental gene regulatory network to filter the propagated noise in both the ON and OFF-states. Our sub-network analyses with *daf-12* and *lin-42* input shows that IFFL are effective in filtering the propagated noise in UNC-5. In other words, with layers of FFLs, it is possible to ensure the reliable target gene expression with minimal propagated noises.

The PFL effect we observed is in line with literature reports showing in increased response time and reduced propagated noise^50^. In the current study, the auto-PFL on *blmp-1* maintains the robust and stable steady state in BLMP-1 until the DRE-1 starts to express. The auto-PFL ensures the BLMP-1 ON-state in a low-noise state and prevents precocious DTC turning. This auto-PFL is coupled with other regulation of *blmp-1* with an OR gate. As a result, the OFF-state may be easily perturbed from an upstream fluctuation in any of its regulators. However, this amplified intrinsic noise in the BLMP-1 OFF-state was largely filtered by the post-transcriptional regulation of DRE-1, and thus the network remains robust to the noise propagated from *blmp-1* and its upstream genes.

Although cells are able to manage noise during development, *blmp-1;daf-12* mutant with an incomplete regulatory network shows a heterogeneous phenotype. Deterministic expression of UNC-5 in this mutant shows a plausible precocious DTC migration. The stochastic simulation in the current study offers a molecular level of understanding for this variability in this system. The mild reduction in the steady-state level of ON-state in UNC-5, and the propagated and intrinsic noises, lead to the heterogeneous mutant phenotype.

Noise filtering effects can be studied by analytical approaches such as a mathematical derivation for the noise-filtering capacity in the frequency domain. These works offer a general understanding, but a linear regulation function is often needed^35^. We employ nonlinear gene regulation function, the Hill function, in the model, with a goal in deriving experimentally relevant insights. Therefore, with a good number of general parameter sets, we study the change of noises in the distribution of FFs. Our approach still offer rather general ground for the observation and for drawing conclusions, without loosing the connection to the biological reality in the model.

In the parameter setting, we fixed the maximum steady-state value for all the proteins as 272 particles per cell. This number is on the lower end, given that experimental works reported 10^2^–10^5^ copies per cell for transcription factors in eukaryotes^51^,^52^. A higher particle number implies a lower level of intrinsic noise. However, because we did not include any other noise source, such as the global noise, and noise propagated from components outside of the network considered, we believe that the setting is a practical compromise that allows us to capture the essential part of the realistic fluctuations.

## Conclusion

We studied the noise propagation in a development network of *C. elegans* that controls the turning time of the DTC. The DTC guides the gonad development, and its turning is well regulated in normal development. The network we studied has multiple IFFL. We found that IFFL can filter propagated noises regardless of the states in the input genes. We also found that the noise-filtering property of an IFFL can be largely derived from that of the individual FFLs. The auto-PFL of *blmp-1* is also helpful in maintaining the desirable activity of *blmp-1* and decreasing the propagated noises but may enhance intrinsic noise of the genes involved. We found that the degradation regulation of DRE-1 also plays a crucial role in reducing the propagated noises in the final target gene UNC-5. Our analyses offers insights into the mechanisms behind the precise DTC migration controls in space and time that are difficult to obtain experimentally. The observation that IFFL can filter both ON and OFF-state noises also allows for better inference from network structures to noise-filtering properties.

## Methods

### General computational settings

As shown in Fig. 1a, the current network has three input genes (*daf-12*, *lin-42* and *dre-1*) and three intermediate and downstream genes (*blmp-1*, *lin-29* and *unc-5*), with their roles in DTC migration experimentally validated. There are other closely related genes and proteins that were not included in the current work, such as *unc-6*, *daf-9*, *daf-16* and *kin-20*. UNC-6 is the netrin guidance cue, which is secreted earlier than the expression of the UNC-5 receptor^53^. DTC migration occurs when the *unc-5* is express^38^. Therefore, UNC-6 expression does not determine the DTC turning and it is not included in the current work. DAF-12 is activated by the binding of dafachronic acid (DA), a steroid hormone synthesized by the enzyme DAF-9^54^. Therefore, DAF-9 is an upstream of DAF-12, which we chose not to include in the model. Previous studies reported that DAF-12 together with DAF-16 regulates the longevity and dauer formation in *C. elegans*^55^. KlN-20 works with lin-42 to regulate the seam cell development^56^. The role of *daf-16* and *kin-20* in DTC migration is unknown.

The duration of current simulations is 20 hr, where 0 to 8, 8 to 16, 16 to 20 hr represents L2, L3, early L4 developmental molt stages respectively. Among the three inputs, *daf-12* is turned ON from the 8^th^ hour and *dre-1* from the 12^th^ hour. *lin-42* has a dynamic, oscillatory expression pattern, and it is in an ON-state from 0.5 to 5 hr and 8.5 to 12 hr in the present work. In the wild type, UNC-5, the key regulator of DTC turning, should start expressing in the mid to late L3 stage, or between 12 and 14 hr. In testing for mutant phenotypes, precocious phenotype should have UNC-5 expressed before 12 hr. For retarded phenotype, UNC-5 should express in L4 stage, which is after 16 hr. If the UNC-5 is not expressed even at 20 hr, we also consider it as a retarded phenotype. The experimental data show that single mutants with *lin-29*, *daf-12* show a wild-type phenotype, and *blmp-1* shows a precocious phenotype. Double mutants including *lin-29;daf-12*, *lin-29;dre-1*, *dre-1;daf-12* show a homogeneous retarded phenotype. Double mutant *blmp-1;daf-12* shows a mixture of precocious and wild-type phenotypes. The complete list of genotypes and their phenotype of DTC turning that we use in this computational study is given in Table 1 37.

We first screen for parameters that can generate UNC-5, LIN-29 and BLMP-1 expression in the wild type by solving the deterministic ordinary differential equations (ODEs). Since we aim to draw general conclusions for noise-filtering properties from the structure of the network, we chose not to further screen for further properties and tested for most of the noise-filtering properties with different network structures with those that can generate wild-type dynamics. A randomly chosen collection of 1000 parameter sets was used in these tests. We further screened for parameter sets that passed the UNC-5 expression in six homogeneous mutants, for testing the dynamics and noises of the mutant. We also screened for parameters with stochastic simulation for homogeneous and heterogeneous phenotypes and tested for the behavior of heterogeneous phenotypes. The details of the deterministic and stochastic stimulation are given in the following sections. All simulations were performed with both Matlab 2010b (The MathWorks Inc. (Natick, MA)) and Octave version 3.8.1^57^.

### Deterministic simulations

Three input genes (*daf-12*, *lin-42*, *dre-1*) are regulated by simple activation and degradation effect as follows.


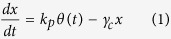


where *x* represents the protein concentration, or the amount of protein per cell (particle numbers in this work), which can be [DAF-12], [LIN-42] or [DRE-1], *k*_*p*_ is the maximum production rate, *γ*_*c*_ is the degradation rate, and *θ*(*t*) describes the regulatory activity for the gene, with 1 indicating that the gene is turned on and 1/4 means it is turned off, or,


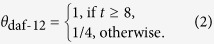


with *daf-12* as an example. For *lin-42*, it is active when *t* = 0.5–5 and *t* = 8.5–12. For *dre-1* it is active when *t* ≥ 12.

In the present work, all components except for *blmp-1* are assumed to degrade at the same rate, *γ*_*c*_. Another simplification we used is to keep a fixed ratio for *k*_*p*_/*γ* at 272, which is the steady-state amount for the gene expression. In the current simulation, we searched for the degradation rates, and then derive *k*_*p*_ from it.

For genes that are regulated by other components in the system, *θ*(*t*) is replaced by by the sum of a basal transcription *α* and a regulated term that varied according to its upstream amount. For example, for *lin-29*, which is regulated by LIN-42 and BLMP-1, is described as follows:


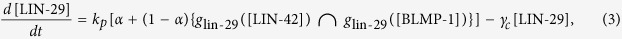


where *α* represents the basal expression which is set to 10% of *k*_*p*_. We used the Hill function for gene regulation, with the activation function being


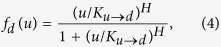


and the repression function as,


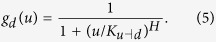


Parameter *H* controls the steepness, which is fixed to 3 throughout the present work. *K* defines the expression of an upstream gene required to half-activate or repress the downstream gene. With more than one signal regulating the expression of a downstream gene, the two signals integrated with a specific regulatory logic^58^,^59^. We use symbol ∩ to represent the combination of two inputs that is similar to an “AND” gate in Boolean algebra:


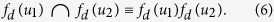


As will be seen below, the combination similar to the “OR” logic gate is denoted with symbol ∪, which is defined as


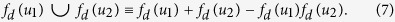


*unc-5* is regulated by DAF-12, LIN-29 and BLMP-1. Its expression is described as,


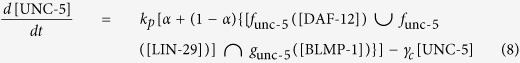


BLMP-1 has post-transnational regulation by DRE-1^42^. Therefore there are two different degradation rates for BLMP-1: a basal rate and an additional rate from DRE-1. The DRE-1 degradation effect is through a Hill function. The ODE of BLMP-1 is as follows:


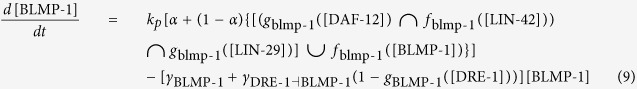


where *γ*_BLMP-1_ is basal degradation rate of BLMP-1, *γ*_DRE-1-|BLMP-1_ is the degradation rate of BLMP-1 by DRE-1.

For BLMP-1, LIN-29 and UNC-5, the relative basal expression strength *α* is kept at 10% of the maximum expression rate. For the input genes the corresponding basal expression was set at 25% in the present work. Without upstream components, input genes are not under the propagated noise, and thus we believe that increasing the basal expression is a convenient way to include propagated noise, especially for the OFF-state.

We adopted the Euler method to numerically propagate the ODEs^60^. This is for consistency with the stochastic simulation, where the Euler-Maruyama method is used^61^,^62^,^63^.

### The regulatory logic combination and their robustness

In the DTC-turning network, the three downstream genes (*lin-29*, *unc-5* and *blmp-1*) are controlled by more than one upstream regulator. Exactly how the upstream signals combine and control these genes are unknown. In setting up the mathematical model, the only requirement is to generate results that are similar to experimental data.

*lin-29* is regulated by LIN-42 and BLMP-1. The combination may be either the AND- or the OR-gates. Since LIN-42 is periodic in expression, and LIN-29 was shown to be expressed only at L3 stage^37^, it is reasonable to assume the AND-logic as,


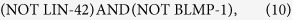


as seen in Equation (3).

*unc-5* is activated by DAF-12, LIN-29 and repressed by BLMP-1. Experimental evidence shows that UNC-5 is expressed in *daf-12* and *lin-29* single mutants. As well BLMP-1 represses UNC-5 expression in early stages. So we can assume that UNC-5 is expressed when DAF-12 or LIN-29 exists, and at the same time BLMP-1 has to be low. In our notation, the logic combination is


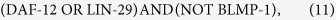


which best fits the experimental evidence. (Equation (8))

*blmp-1* is regulated by four regulatory inputs, DAF-12, LIN-42, LIN-29 and itself. Single mutant *blmp-1* shows a homogeneous phenotype, whereas the *blmp-1;daf-12* double mutant shows a heterogeneous phenotype (Table 1). From these experimental observations, it is not straightforward to conclude with one logic combination. So we further analyzed eight different combinations of logic gates at BLMP-1 (Supplementary Table S1). We choose the eight logic combination based on our understanding of the experimental system. All eight logic combinations were scanned using 1.48 × 10^10^ parameters for generating the wild type and 6 homogeneous phenotypes as we described previously. Among all logic gates tested, we found the following combination has the largest number of parameters that can reproduce both wild type and six homogeneous mutants,


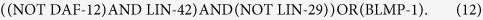


This result means that the network with this logic combination is the most robust in terms of tolerating different parameter values, and it is the one we used for the present study.

### Stochastic simulations

Typical stochastic simulations can be performed with the Gillespie’s algorithm^64^,^65^ or the Langevin’s equation^66^. By setting a large leaping time that allows many fundamental reaction steps, the Langevin’s equation propagates with the expected progress and a Gaussian noise to account for the fluctuations. For typical reactions that do not involve gene expression, such as protein degradation or protein-protein interactions, the statistics follows Poisson statistics, and therefore, the variance of the fluctuation equals the mean propagation in each leap^66^:


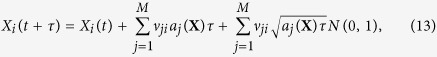


where *v*_*ji*_ is the stoichiometry number for species *X*_*i*_ in reaction *j*. In other words, it is the number of molecules consumed (negative) or created (positive) when reaction *j* happens. *a*_*j*_(**X**) is the reaction propensity, or the reaction rates, when the systems has {**X**} particle number combination. *N*(0, 1) is the Gaussian random number with zero mean and unit variance.

Genes are expressed in bursts^6^,^67^. In each expression event, the number of mRNA or protein transcripts produced is not one, but a number that is fluctuating in each burst. The Poisson statistics behind the Langevin equation as in Equation (13) is no longer valid, and it should be modified for gene expression channels. In ref. 43, it is shown that the FF in gene expression becomes 2*b* + 1 where *b* is the mean burst size, or 2*b* if *b* ≫ 1. For a step size *τ* in propagation, with the mean burst frequency as *k*_*b*_, the mean protein production is *k*_*b*_*bτ*. The variance for protein burst production becomes 2*b* times the mean, 2*k*_*b*_*b*^2^*τ*. Thus, the following terms are used in the Langevin equation for gene expression,


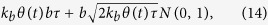


for the input genes. For the three regulated genes, *θ*(*t*) is replaced by [*α* + (1 − *α*)(Hill regulation functions)]. We note the term *k*_*b*_*b* is the maximum production rate, which is equivalent to *k*_*p*_. In our simulation, we fixed the value of *b* (as 16), and set


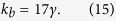


The maximum protein production rate 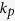 becomes


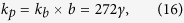


leading to the maximum steady-state protein number as


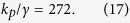


### Search for parameter sets with wild type and homogeneous phenotypes

In searching for suitable parameters, we determined a set of parameter values and systematically scanned through all the combinations. In the scanning, we test for UNC-5, LIN-29 and BLMP-1 expression that can mimic the wild type and UNC-5 expression in various mutant phenotypes. In this stage, we propagated the expression dynamics without any noise. All regulatory interactions such as activation and inhibitions in gene expression are described with Hill functions. Since we kept the maximum steady-state value for all genes, *k*_*p*_ was derived once the degradation rates (*γ*’s) are known. We systematically scanned the degradation rates and activation/repression threshold values over a range of parameters. For threshold (*K*) values, 6 different values were roughly equally spaced between the lower and upper values in Table 2, which were chosen to be similar or lower than the maximum protein level (272 copies per cell). The first-order degradation rates of DAF-12, LIN-42, LIN-29, DRE-1, UNC-5 are assumed to be the same, but separate from that of BLMP-1. BLMP-1 has a post-translational regulation by DRE-1^42^. Therefore, two degradation rates for BLMP-1, a basal rate and an additional rate from DRE-1 were separately chosen. As listed in Table 2, values of degradation rates were equally spaced by a factor of 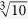. I.e. there are three scanned values in every order of magnitude scanned. For genes other than *blmp-1*, one of the five values listed in Table 2 was chosen first such that the time it takes to change from steady-state to another, when the upstream regulation is varied, is similar to or faster than 1 hour, as they have to be shorter than developmental time scales, with each larva stage being 8 hours. The maintenance of BLMP-1 activity even in late L2 stage, when its activator *lin-42* is low, implies that the degradation of BLMP-1 may be slow. Therefore, we scanned *γ*_BLMP−1_ relative to *γ*_*c*_ with a factor that is smaller or equal to 1, i.e.,


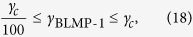


with the factors relative to *γ*_*c*_ scanned (as listed in Table 2). For BLMP-1 degradation due to DRE-1, we assume that it is similar to *γ*_*c*_ in their orders of magnitude. Thus we set


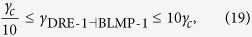


with the factors relative to *γ*_*c*_ scanned (as listed in Table 2). Using a deterministic approach, we scanned approximately 1.48 × 10^10^ parameters to check whether they can reproduce the wild type and six homogeneous mutant phenotypes (i.e. wild type or precocious or retarded). During parameter selection, in the wild-type setting, we first require that UNC-5, LIN-29 should be turned ON, and BLMP-1 should be turned OFF in late L3 (*t* = 12 − 14 *hr*), Second, the expression of UNC-5 should be > 66% of the maximum steady-state level. Third, the difference in the OFF-state and the ON-state expression of UNC-5, LIN-29 and BLMP-1 should be at least 66% of its ON-state steady state. We obtained 3.08 × 10^7^ parameter sets with this criteria, and 1000 of them were randomly chosen for our study on network structure and their noise-filtering properties.

For parameter sets that reproduce six mutant phenotypes (*blmp-1*, *lin-29*, *daf-12*, *lin-29;dre-1*, *lin-29;daf-12*, *dre-1;daf-12*), further looked for an early (*t* < 12 *hr*), normal, or late (*t* > 16 *hr*) switch in UNC-5 expression according to the mutant phenotypes. In simulating mutants, the gene that is loss-of-function is simply set to zero in the mathematical model, and the propagation of dynamics proceeds without any other change in the model setting. We have obtained 5.35 × 10^6^ parameter sets at this stage, and 1000 of these were used in the study of homogeneous mutants *lin-29* and *daf-12*.

To support the generality related to noise-filtering properties of the system, we have also performed an alternate parameter selection strategy using random search (Monte Carlo simulation). Here we set independent production and degradation rates for each gene within a parameter range as shown in supplementary Table S2, that include the parameters used in systematic scan mentioned above. Again we screen for parameter sets that can reproduce the wild type using deterministic ODE as in systematic parameter scanning. We confirm that the noise filtering capacity of subnetworks through IFFL and PFLs, built from this more general random sampling of parameters (Supplementary Fig. S6–8) is similar to the results derived from the complete systematic scanning. Detailed discussion on justifying the model setting used in the present work, is included in the supporting information.

### Simulation for heterogeneous phenotypes

After obtaining parameter sets that can reproduce the wild type and six homogeneous mutant phenotypes in the deterministic simulation, stochastic simulation were used to further narrow down the parameters that can reproduce the wild type, six homogeneous and one heterogeneous mutant phenotypes (*blmp-1;daf-12*) observed in experiments. Since now the UNC-5 expression is stochastically fluctuating, one cannot directly set a threshold for UNC-5 for DTC turning. Instead we assume that the DTC turning requires downstream effects that may be propagated or accumulated in a slow time scale, and we integrated UNC-5 level for 3.5 hr and obtained the cumulative UNC-5 signals. The threshold value for the cumulative signals was determined as the integrated value of the wild-type model in the deterministic condition, the “standard” condition, at the desirable DTC turning time (14 hour). The time when the integrated value from stochastic simulation passing this threshold is modelled as the DTC turning time.

## Additional Information

**How to cite this article**: Chepyala, S. R. *et al*. Noise propagation with interlinked feed-forward pathways. *Sci. Rep*. **6**, 23607; doi: 10.1038/srep23607 (2016).

## Supplementary Material

Supplementary Information

## Figures and Tables

**Table 1:** Experimental observation of DTC turning in different genotypes^37^.

Genotype	DTC turning phenotype
*blmp-1(s71)*	precocious
*lin-29(n546)*	wild type
*daf-12(rh61rh411)*	wild type
*lin-29(n546);dre-1(dh99)*	retarded
*lin-29(n546);daf-12(rh61rh411)*	retarded
*dre-1(dh99);daf-12(rh61rh411)*	retarded
*blmp-1(s71);daf-12(rh61rh411)*	precocious (31%) wild type (43%)

**Table 2:** Range of parameters scanned.

Parameter	unit	Parameter values						
*K*^a^	Particle numbers	80	125	170	215	260	300	
*γ*_*c*_^b^	hour^−1^	0.775	1.67	3.6	7.75	16.7		
*γ*_BLMP-1_^c^	*γ*_*c*_^c^	0.01	0.04642	0.02154	0.1	0.4642	0.2354	1
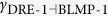 d	*γ*_*c*_^d^	0.1	0.4642	0.2354	1	4.642	2.354	10

^a^For all threshold values, including *K*_DAF-12→UNC-5_, 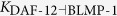, 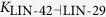, *K*_LIN-42→BLMP-1_, 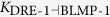, 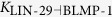, *K*_LIN-29→UNC-5_, *K*_BLMP-1→BLMP-1_, 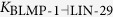, and 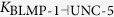.

^b^Degradation rate for all proteins except BLMP-1.

^c^Basal degradation rate of BLMP-1, scanned relative to the *γ*_*c*_ value chosen.

^d^Degradation rate of BLMP-1 by DRE-1, scanned relative to the *γ*_*c*_ value chosen.
