# Supplementary material for: Noise propagation with interlinked feed-forward pathways
Source: Sci Rep. 2016 Mar 31;6:23607. doi: 10.1038/srep23607 (PMC4814832; doi:10.1038/srep23607)
Supplement: Supplementary Information [file srep23607-s1.pdf]

Supplementary Material for: Noise propagation with interlinked feed-forward pathways

Surendhar Reddy Chepyala[1,2,3], Yi-Chen Chen[2,4], Ching-Cher Sanders Yan[2], Chun-Yi David Lu[4], Yi-Chun Wu[5,6,7,8,9,*], and Chao-Ping Hsu[2,5,*]

[1]Bioinformatics Program, Taiwan International Graduate Program, Institute of Information Science, Academia Sinica, Taipei, Taiwan.
[2]Institute of Chemistry, Academia Sinica, Taipei, Taiwan.
[3]Institute of Biomedical Informatics, National Yang-Ming University, Taipei, Taiwan.
[4]Department of Chemistry, National Taiwan University, Taipei, Taiwan.
[5]Genome and Systems Biology program, National Taiwan University, Taipei, Taiwan.
[6]Institute of Molecular and Cellular Biology, National Taiwan University, Taipei, Taiwan.
[7]Department of Life Science, National Taiwan University, Taipei, Taiwan.
[8]Center for Systems Biology, National Taiwan University, Taipei, Taiwan.
[9]Research Center for Developmental Biology and Regenerative Medicine, National Taiwan University, Taipei, Taiwan.
[*]Chao-Ping Hsu, Email: cherri@sinica.edu.tw, Fax: 886-2-2783-1237, Yi-Chun Wu, Email: yichun@ntu.edu.tw, Fax: 886-2-3366-5248

Suplimentary Information

Supporting Results

Additional results

In this supplementary material, we offer additional results for the interested readers. We first include the results of subnetworks with positive feedback loops (PFLs) for propagated noises on BLMP-1 and LIN-29 (Fig. S1), which helps to understand the final noise on UNC-5 as discussed in the main text. In these tests, the noise was included for DAF-12 (Fig. S1a) or LIN-42 (Fig. S1b), and the noise effect on BLMP-1 and LIN-29 were shown. It is seen that auto-PFL on BLMP-1 filters the noise asymmetrically in BLMP-1 with no noise observed in ON-state (subnetwork D and subnetwork T). Relative noise levels were observed in LIN-29 in the both sub networks. PFL with mutual inhibition shows a reduced noise levels in both the states of BLMP-1 with *daf-12* input (subnetwork S). However in subnetwork T, with *lin-42* input, PFL with mutual inhibition shows a decreased noise in BLMP-1 OFF state and LIN-29 ON state with a mild increase in corresponding opposite states. One reason for mild increase in noise is due to the cFFL connecting them showing asymmetric noise behavior.

The effects of intrinsic noise of BLMP-1 and LIN-29 is included in Fig. S2. In these tests, only the noises of BLMP-1 and LIN-29 were included. It is seen that the mutual inhibition between *blmp-1* and *lin-12*, a PFL leads to very similar distribution in the Fano factor. However, adding the auto-activation of *blmp-1* (auto-PFL) leads to a large increase in the FF of both BLMP-1 and LIN-29, especially in the OFF-state of LIN-42. Again the increased noise due to the asymmetric response time of auto-PFL and lower auto-activation threshold of *blmp-1* that we have discussed in the main text.

The distribution of the activation/repression threshold values obtained are included in Fig. S3. It is seen that auto-PFL for the regulation from BLMP-1 has a strong preference over the lower values, as discussed in the main text. The two other threshold values for BLMP-1 are also preferred in the lower values, but most of other K values are not. One possible reason is that the OFF-state expression of DRE-1 reduces the BLMP-1 steady state expression, and the effect is stronger than that for other nodes in the network. With a relatively low BLMP-1 value, the scanned threshold values are also lower compared to those for other nodes.

The trajectories of UNC-5 in wild type and mutants, calculated from a representative parameter set, is as shown in Fig. S4. It is seen that *daf-12* and *lin-29* mutants has a similar activation time for UNC-5, while *blmp-1* mutant has an early activation, corresponding to the precoucious phenotype. The three combined mutation, *dre-1;daf-12*, *lin-29;daf-12*, and *lin-29;dre-1* has

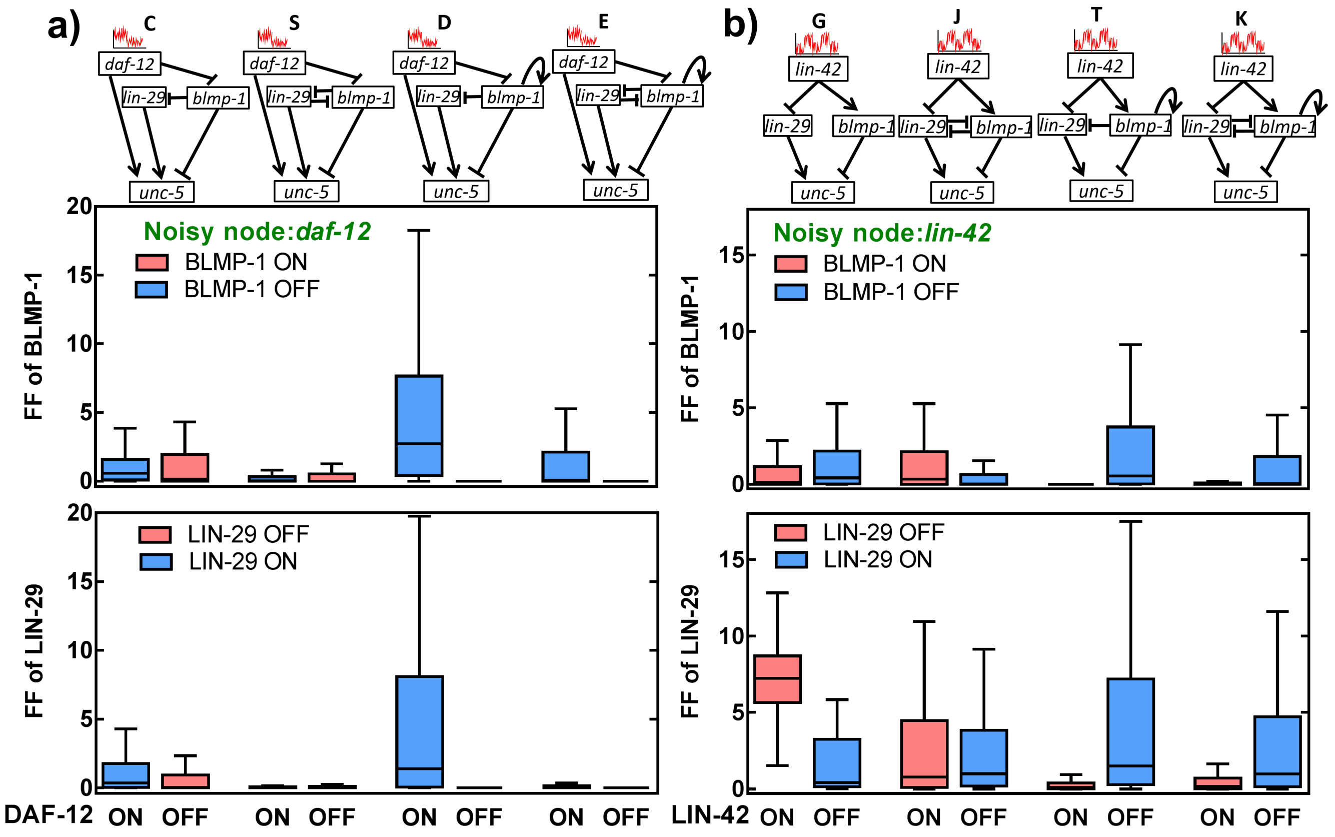

Figure S1. Role of positive feedback loops in buffering the propagated noise on BLMP-1 (upper panel), and LIN-29 (Lower panel). (a) Propagated noise observed while DAF-12 being noisy. (b) Propagated noise observed while LIN-42 being noisy.

no activation in UNC-5, which corresponds to the retarded phenotype. Mutant *blmp-1;daf-12* has an oscillatory activity for UNC-5, which we further study with stochastic simulation.

The phenotype distribution from stochastic simulation, for the genotypes that are not included in the maintext, is included in Fig. S5. We observe that DTC turning distribution in *lin-29* and *daf-12* single mutants is slightly wider compared to WT (Fig. 7c in main text). This result correlates with the higher noise we observed in these mutants at the time of DTC turning (late L3 stage) (Fig 6a in main text). Single mutation in *blmp-1* results in a precocious distribution where DTC turning observed at early L3 stage and with some at late L2. The three double mutants, *dre-1;daf-12*, *lin-29;daf-12*, and *lin-29;dre-1* do not show UNC-5 expression even at 20 hr.

In Table S1 we list the results of testing over the logic combination for the regulation of *blmp-1* from the three upstream genes and an auto-activation. Reported are the fractions of parameter sets that passed the screening of wild type or those also passed the 6 mutants as described in the Method section of the main text. It is seen that logic No.2 has the highest rates in both cases, and therefore, it is used for all the computational analysis in the present work.

Alternative random screening for parameters

We have tested with a random search (Monte Carlo simulation) with independent production and degradation rates for each gene(Table S2), and it was repeated with two different burst sizes. Again we screen for parameter sets that can reproduce the wild type using deterministic ODE by requiring a clear distinction between the ON and OFF state level as described in Method section(page 15) of the main text.

We confirmed that the noise filtering capacity of subnetworks through IFFL and PFL, built from this more general random sampling of parameters, is similar to the conclusions derived from the complete scanning performed and reported in the main text. From Fig.S6, S7 and S8, it is seen that the changes from case to case remain the same, but the observed FF levels are higher with a wider distribution, simply due to the different steady state obtained from various production rates sampled. Similar results are also observed (Fig.S9, S10 and S11) when the burst size was set at 64.

Table S1. Logic combinations tested at *blmp-1* node

Number	Logic	Fraction of parameter sets that can reproduce wild type	Fraction of parameter sets that can also reproduce 6 homogeneous mutants
1	(($\overline{\text{DAF-12}}$[a]AND LIN-42) AND $\overline{\text{LIN-29}}$) AND BLMP-1	1.377×10^{-8}	0
2[b]	(($\overline{\text{DAF-12}}$ AND LIN-42) AND $\overline{\text{LIN-29}}$) OR BLMP-1	2.079×10^{-3}	3.612×10^{-4}
3	(($\overline{\text{DAF-12}}$ AND LIN-42) OR $\overline{\text{LIN-29}}$) AND BLMP-1	9.968×10^{-4}	1.718×10^{-4}
4	(($\overline{\text{DAF-12}}$ OR LIN-42) AND $\overline{\text{LIN-29}}$) AND BLMP-1	9.384×10^{-5}	1.386×10^{-5}
5	(($\overline{\text{DAF-12}}$ OR LIN-42) OR $\overline{\text{LIN-29}}$) AND BLMP-1	1.145×10^{-3}	2.017×10^{-4}
6	(($\overline{\text{DAF-12}}$ AND LIN-42) OR $\overline{\text{LIN-29}}$) OR BLMP-1	1.050×10^{-3}	4.559×10^{-5}
7	(($\overline{\text{DAF-12}}$ OR LIN-42) AND $\overline{\text{LIN-29}}$) OR BLMP-1	1.105×10^{-3}	1.203×10^{-4}
8	(($\overline{\text{DAF-12}}$ OR LIN-42) OR $\overline{\text{LIN-29}}$) OR BLMP-1	7.171×10^{-4}	2.858×10^{-5}

[a] The overline indicates NOT logic operation. Namely it is true when the protein is low in activity.
[b] Number 2 is the one employed in the present work, since the number of parameter sets passing both criteria (Wild type and 6 homogeneous mutants) is the largest.

Table S2. Range of parameters used in random scan

Parameter	Unit	Lower value	Upper value
γ_c [a]	hour^{-1}	0.1	20
$\gamma_{\text{BLMP-1}}$ [b]	min γ_c[b]	0.001	1
$\gamma_{\text{DRE-1}\dashv\text{BLMP-1}}$	hour^{-1}	0.01	200
k_p [c]	hour^{-1}	10	20000
K [d]	Particle numbers	regulator OFF-state level	regulator ON-state level

[a] Degradation rate for all proteins except BLMP-1. The value for each component was independently scanned in this test.
[b] Basal degradation rate of BLMP-1, scanned relative to the minimum of γ_c value chosen for DAF-12, LIN-42, DRE-1, LIN-29.
[c] Production rate for all proteins, each independently scanned.
[d] For all threshold values, including $K_{\text{DAF-12}\rightarrow\text{Unc-5}}$, $K_{\text{DAF-12}\dashv\text{BLMP-1}}$, $K_{\text{LIN-42}\dashv\text{LIN-29}}$, $K_{\text{LIN-42}\rightarrow\text{BLMP-1}}$, $K_{\text{DRE-1}\dashv\text{BLMP-1}}$, $K_{\text{LIN-29}\dashv\text{BLMP-1}}$, $K_{\text{LIN-29}\rightarrow\text{UNC-5}}$, $K_{\text{BLMP-1}\rightarrow\text{BLMP-1}}$, $K_{\text{BLMP-1}\dashv\text{LIN-29}}$, and $K_{\text{BLMP-1}\dashv\text{UNC-5}}$.

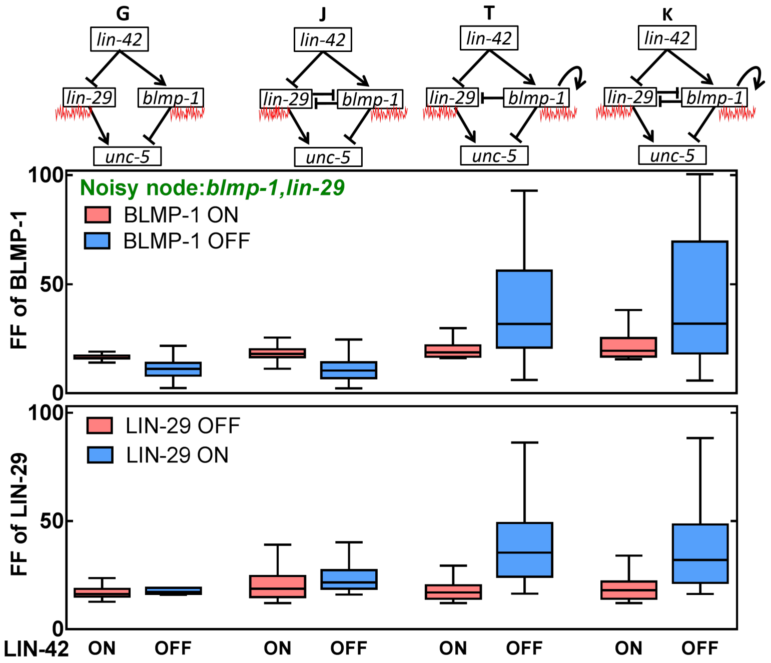

Figure S2. Auto-PFL increses the intrinsic noise. Intrinsic noise observed in BLMP-1 (upper panel), and LIN-29 (Lower panel) while BLMP-1 and LIN-29 are being noisy.

Additional remarks on parameter settings

The time scales of changes are mainly determined by the degradation time (γ's).[1,2] In a dynamic system for developments such as the currently studied DTC cell migration, gene expression changes at various levels through the development stages, measured in hours. Therefore, the degradation time should allow such change to take place with a time scale of hours, which set a lower bound for γ's. Very large γ's allow fast switch for the genes, which would allow the same conclusion in modeling the simulation, but very fast switches would make numerical propagation of the ODE's more time-consuming but with essentially the same results, and therefore we pick a range of γ's according to these constraints.

BLMP-1 has additional degradation effect by DRE-1 as observed in experiments[3,4] supporting that BLMP-1 self- degradation rate can be lower than other components in the network. So in all simulations in the present work, we assume BLMP-1 basal degradation is slower than other regulating components such that DRE-1 degradation is effective in simulation and the experimental observations can be reproduced. Again our results showed that the main conclusion on noise-filtering is unaffected as long as it is set to be slower than other components.

The level of gene expression (k_p/γ) has been quantified for many different systems.[5,6] While it is not known for the specific development state that we focused on, we can simply allow it to very across a reasonable range as set by experimental results[5,6] in this Monte Carlo sampling. However, we note that none-dimensionalization allows us to choose arbitrary units for each component for deterministic simulation, as people have published excellent works with this non-dimensional setting.[7,8] Therefore, our deterministic results in the main text is not affected by the setting in the gene expression. The new results from Monte Carlo sampling indicate that the noise-filtering behavior is indeed not affected.

The burst size b has a direct consequence on the size of fluctuation in each component. And the fluctuation is relative to gene expression level, and thus, the gene expression level does have an effect on the noise amplitude. In the main text, we simplified the simulation by using a fixed overall expression level and a fixed burst size. In the supplementary we included new Monte Carlo sampled parameter search with a large variation in the gene expression rates but with a fixed burst size. We choose the latter setting to test simply because the effects of varying both is mainly in the variation of fluctuation (Fano factor) of each gene, which can be equally achieved by varying the expression rates only. The fact that both simulation leads to similar conclusions in noise-filtering indicates that the level of noise (and factors behind such noises) is largely independent to the noise-filtering mechanism. We note that, in an additional test, a change in the burst size (from 16 to 64) leads to the same overall dynamics, the same changes in the fluctuation, and the same conclusions in noise filtering, which further confirms our conclusion here.

The threshold value (K's) of each gene regulation has the same dimension as the corresponding regulator's abundance. For a gene regulation to be effective, it has to be turned ON and OFF as observed and concluded in experiments. Therefore, the

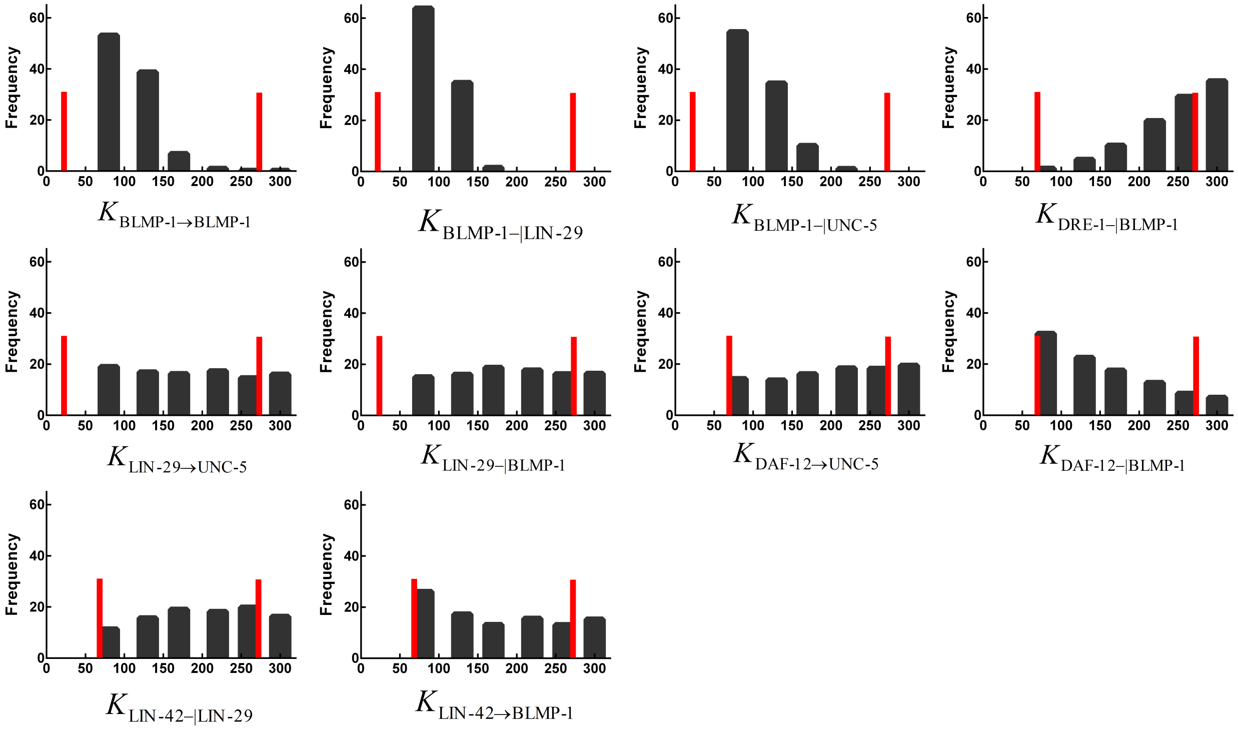

Figure S3. Percentage of activation/repression threshold value that were selected by different regulators while fitting the wild type expression of UNC-5, LIN-29 and BLMP-1. The two red vertical bars indicates lower and higher steady-state values for the regulating component.

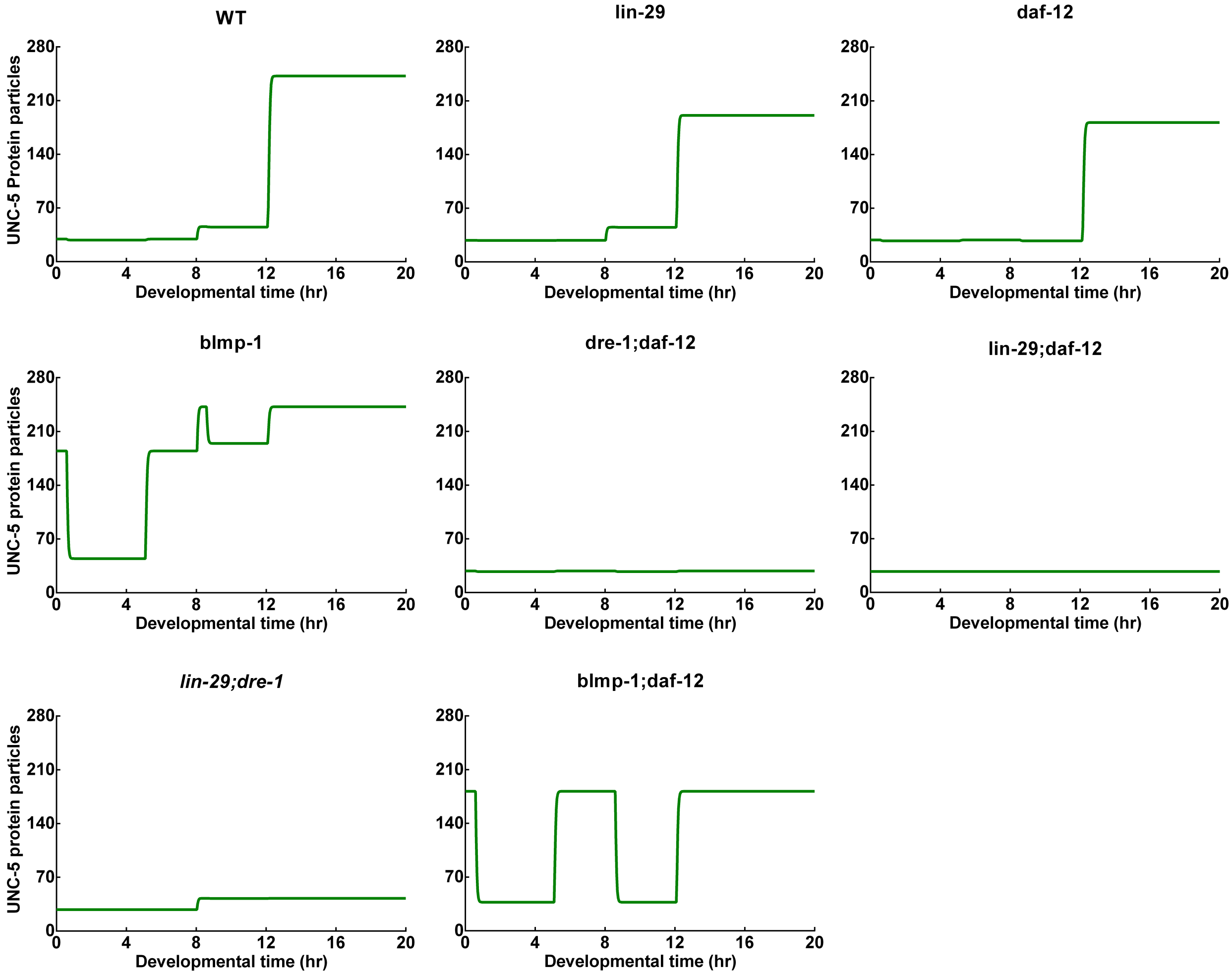

Figure S4. Simulated UNC-5 expression of DTC in wild type and mutants mentioned in Table 1.

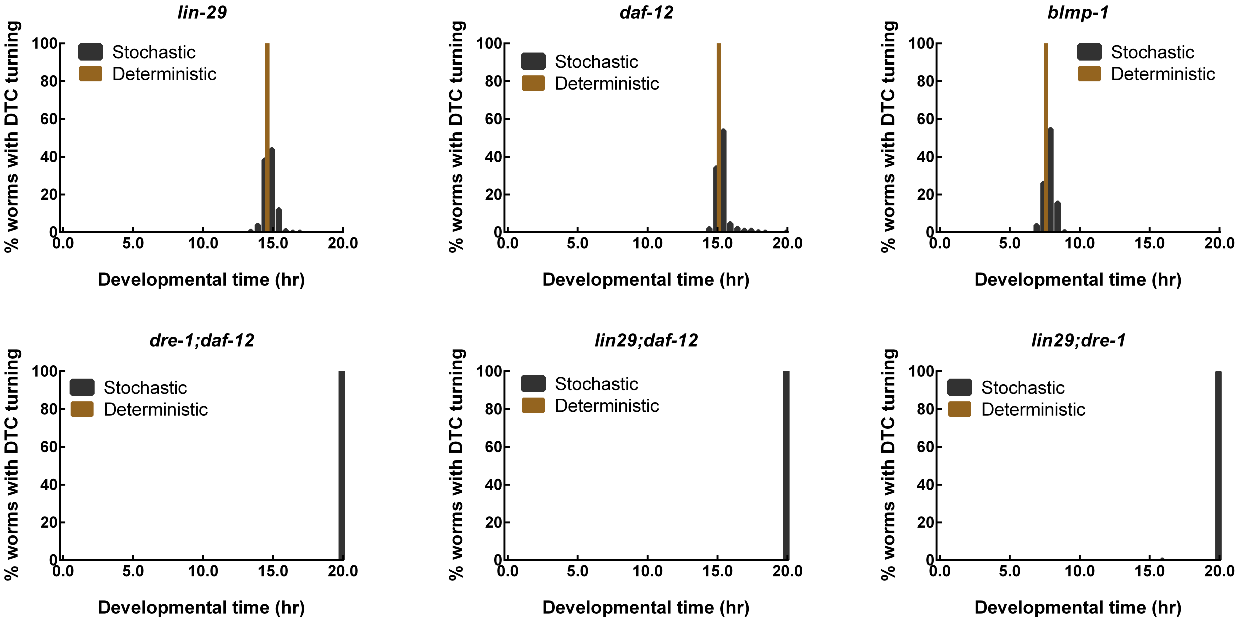

Figure S5. DTC turning time distribution in mutants.

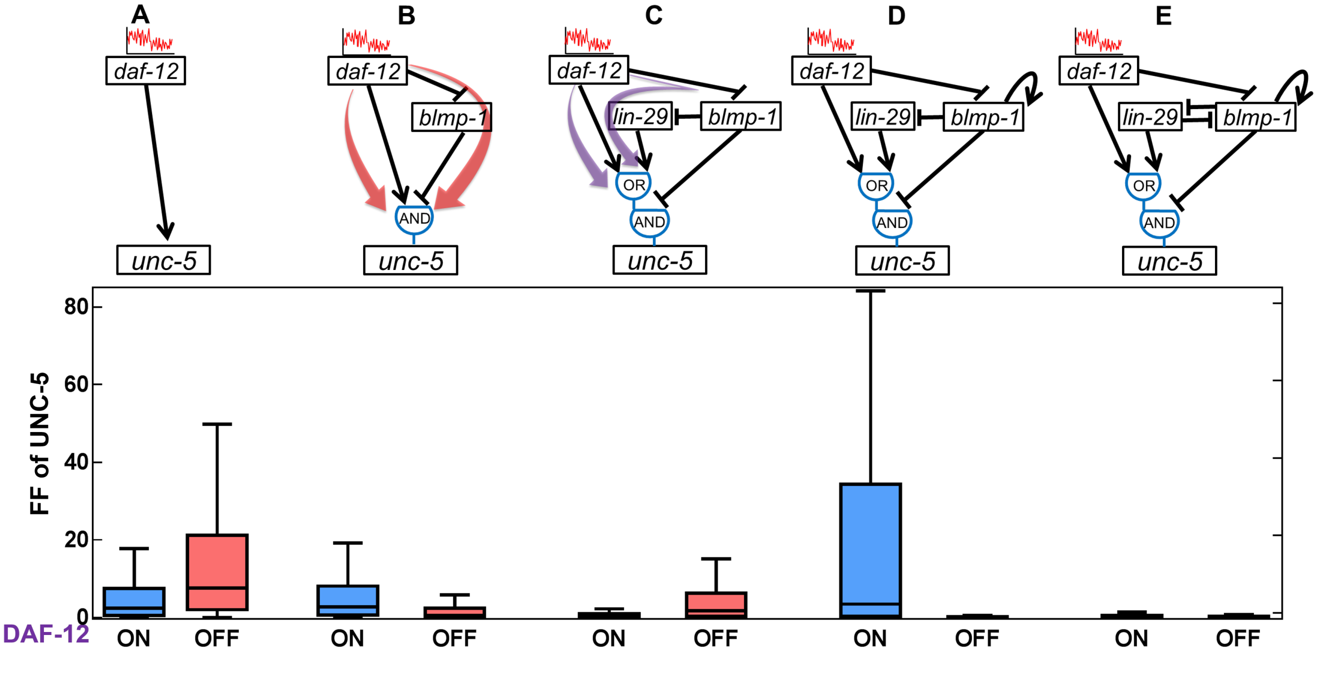

Figure S6. Random screening of parameters showing propagated noises from DAF-12 as observed fluctuation in UNC-5 in different subnetworks as shown on the top. The results were obtained by setting the burst size b as 16.

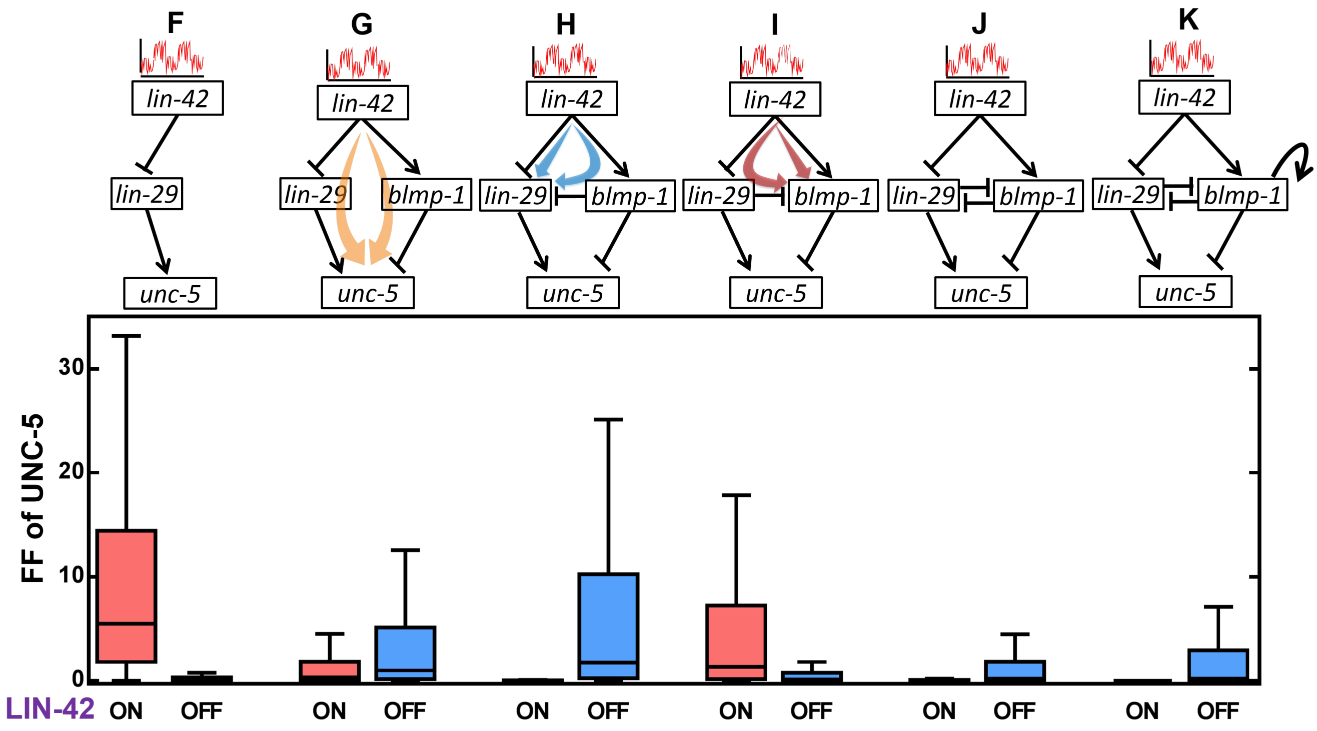

Figure S7. Random screening of parameters showing propagated noises from LIN-42 as observed fluctuation in UNC-5 in different subnetworks as shown on the top. The results were obtained by setting the burst size *b* as 16.

threshold values has been chosen according to the ON and OFF-state level of the regulators in both tests. We note that a wider choice of the threshold values is not that necessary because it would simply create an all-ON (for very small K's) or all-OFF (for very large K's) situation through out the dynamics of development, and such situation means a change in regulation is not possible, which is often not what experimentally observed, and can be ruled out safely.

In the parameter scanning presented in the main text, the combination of all 13 parameters yields 1.48×10^{10}different combination already. In that simulation, we obtained 3.08×10^{7} combinations that pass the wild-type requirements, and a randomly chosen 1000 sets were used for the current work, which offers a very general ground for the conclusions. In the new round of Monte Carlo simulation (with *b* equals 16), the number of independent parameters was increased to 23, with a broader parameter setting, the chance of finding a suitable parameter set dropped from 0.2 % to 0.006 %. Again the same conclusion can be drawn for this new setting.

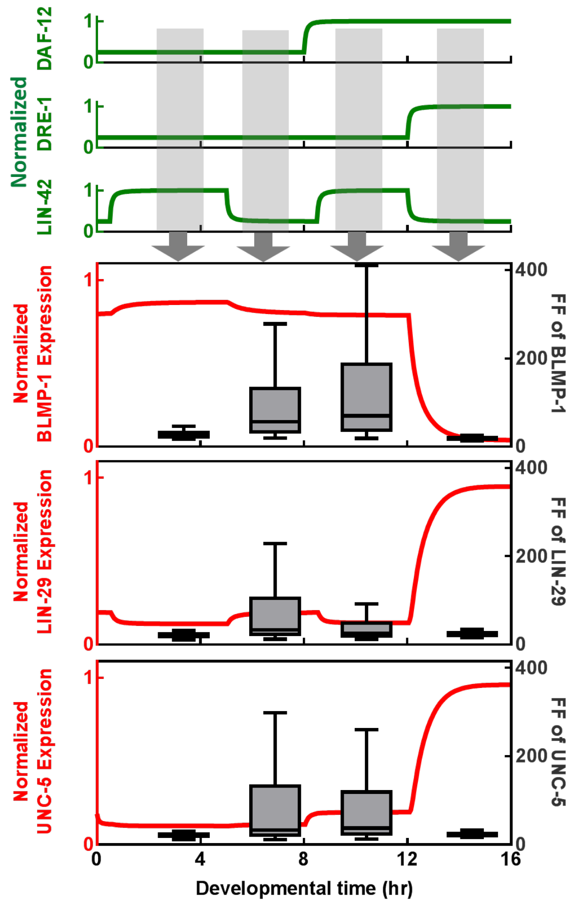

Figure S8. Random screening of parameters showing noise propagation in the complete network, calculated with intrinsic noises included in all nodes. The results were obtained by setting the burst size *b* as 16.

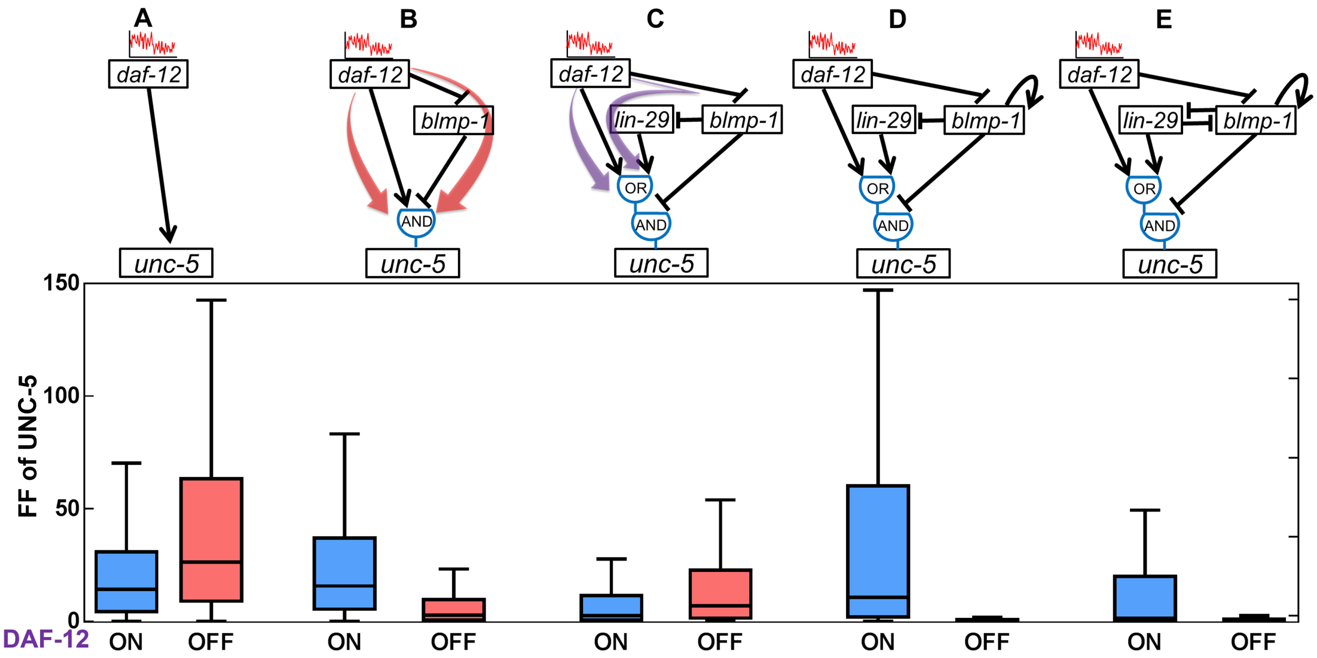

Figure S9. After increasing the burst size, random screening of parameters showing propagated noises from DAF-12 as observed fluctuation in UNC-5 in different subnetworks as shown on the top. The results were obtained by setting the burst size *b* as 64.

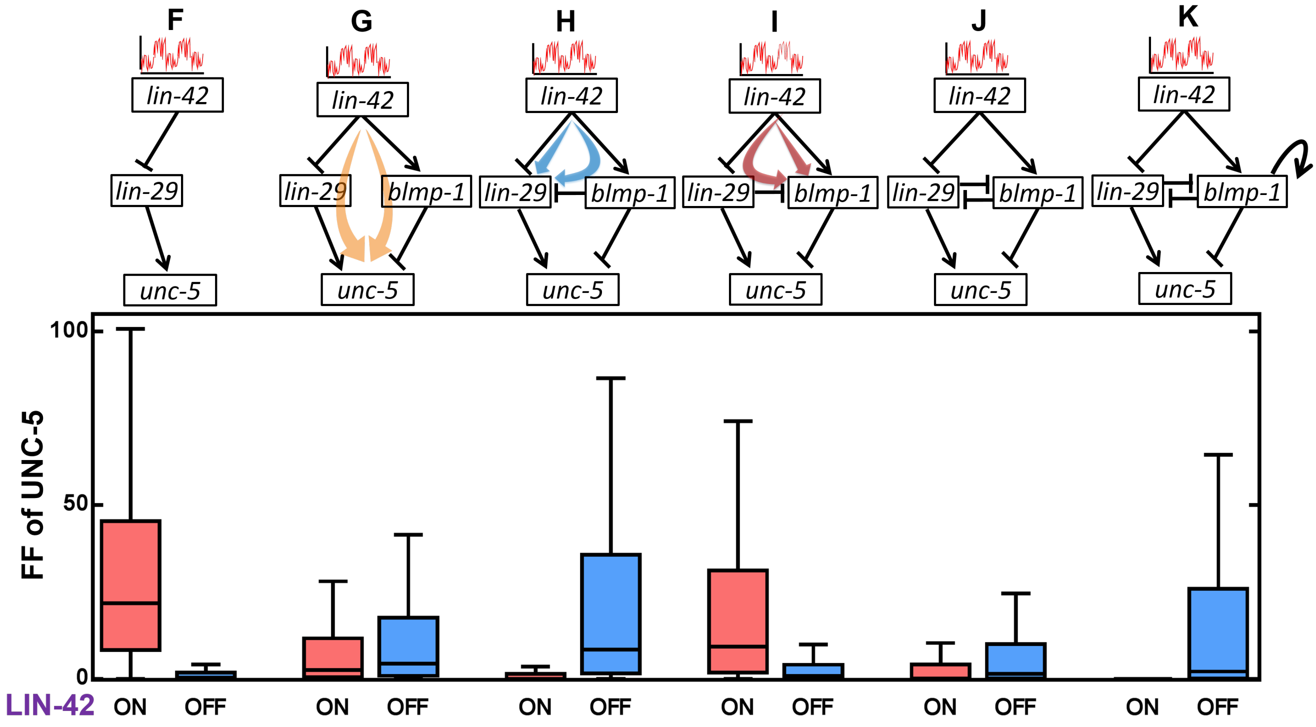

Figure S10. After increasing the burst size, random screening of parameters showing propagated noises from LIN-42 as observed fluctuation in UNC-5 in different subnetworks as shown on the top. The results were obtained by setting the burst size *b* as 64.

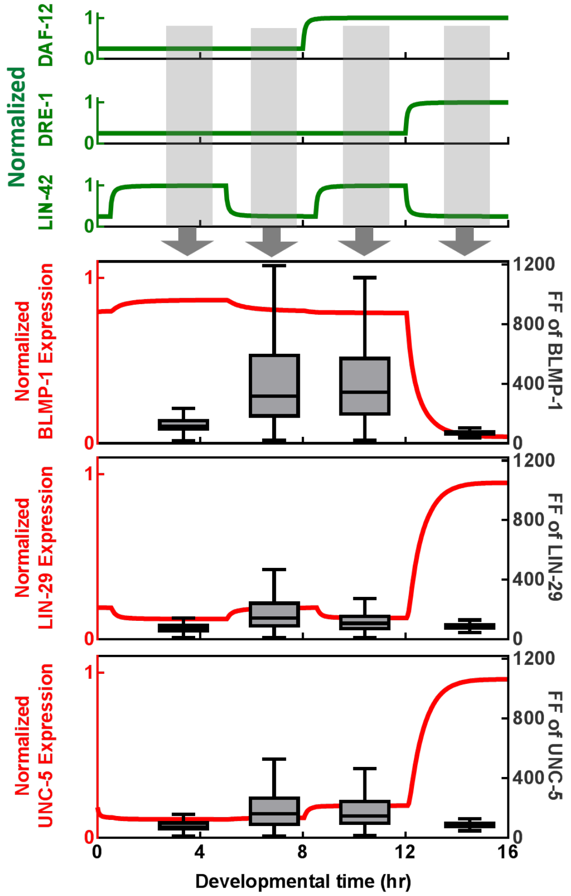

Figure S11. After increasing the burst size, random screening of parameters showing noise propagation in the complete network, calculated with intrinsic noises included in all nodes. The results were obtained by setting the burst size *b* as 64.

References

1. Paulsson, J. Summing up the noise in gene networks. *Nature* **427**, 415–418 (2004).

2. Elf, J. & Ehrenberg, M. Fast evaluation of fluctuations in biochemical networks with the linear noise approximation. *Genome Res.* **13**, 2475–2484 (2003).

3. Horn, M. *et al.* DRE-1/FBXo11-dependent degradation of BLMP-1/BLIMP-1 governs C. elegans developmental timing and maturation. *Dev. Cell* **28**, 697–710 (2014).

4. Huang, T.-F. *et al.* BLMP-1/blimp-1 regulates the spatiotemporal cell migration pattern in C. elegans. *PLoS Genet.* **10**, e1004428 (2014).

5. Milo, R. & Phillips, R. *Cell Biology by the Numbers* (Garland Science, 2015).

6. Biggin, M. D. Animal transcription networks as highly connected, quantitative continua. *Dev. Cell* **21**, 611–626 (2011).

7. Tsai, T. Y.-C. *et al.* Robust, tunable biological oscillations from interlinked positive and negative feedback loops. *Science* **321**, 126–129 (2008).

8. Pomerening, J. R., Kim, S. Y. & Ferrell, J. E. Systems-level dissection of the cell-cycle oscillator: bypassing positive feedback produces damped oscillations. *Cell* **122**, 565–578 (2005).
